# Association of educational environment with the prevalence of myopia: a cross-sectional study in central China

**DOI:** 10.3389/fpubh.2023.1188198

**Published:** 2023-06-15

**Authors:** Wei Peng, Zikang Zhang, Fei Wang, Shaoming Sun, Yining Sun

**Affiliations:** ^1^Hefei Institutes of Physical Science, Chinese Academy of Sciences, Hefei, China; ^2^University of Science and Technology of China, Hefei, China; ^3^The Second Hospital of Anhui Medical University, Hefei, China; ^4^CAS Hefei Institute of Technology Innovation, Hefei, China

**Keywords:** myopia, educational environment, load of studying, risk factors, China

## Abstract

**Purposes:**

This study was to estimate the prevalence of myopia among primary school students in Hefei, China, and evaluate the association of educational environment with myopia.

**Methods:**

This study was a cross-sectional study, and recruited primary school students in grades 1–6. Children underwent a stepwise ophthalmic examination, which included visual acuity and objective cycloplegic refraction to identify children with myopia. Under the guidance of parents, children completed a questionnaire, including gender, region, grade and several indicators related to education. The study analyzed the risk factors by using a logistic regression and assessed feature importance by using a random forest algorithm.

**Results:**

A total of 3,596 primary school students were involved in this analysis, and the overall prevalence of myopia was 27.1%. Gender, grade, education level of the father, education level of the mother, academic level of children, hours of homework per day on weekends, number of after-school tutoring per week and frequency of extracurricular reading were significantly associated with myopia. There was no significant association between the amount of homework per day on school days and myopia after adjusting for covariates. In terms of educational environment, the top 3 factors were academic level of children, homework on weekends and after-school tutoring.

**Conclusions:**

Educational environment with high educational loads was associated with the high prevalence of myopia. Reducing the burden of studying, especially that after class, was an effective way to prevent myopia.

## 1. Introduction

The prevention and control of myopia has been one of the important challenges for public health across the globe. World report on vision from the WHO showed that the overall prevalence of myopia was highest in high-income countries of the Asia-Pacific region (53.4%), closely followed by East Asia (51.6%) (1). Starting from 2018, the National Health Commission of the People's Republic of China has regularly carried out the screening work of myopia by using visual acuity test and non-cycloplegic refraction. The statistics showed that the overall prevalence of screening myopia in children and adolescents aged 6–15 in 2020 was 52.7%, and increased by 2.5 percentage points compared with that in 2019 (2).

The occurrence of myopia has a clear genetic predisposition. Parental myopia (3, 4) and genetic information (5) have been demonstrated to be strongly associated with myopia. However, more and more studies have reached a consensus that the prevalence of myopia is increasing too quickly to simply be due to genetics, and environmental factors play a leading role (6–8). The major environmental factors associated with myopia included near work (9, 10) and outdoor time (11–13). According to a meta-analysis based on randomized controlled trials, outdoor time has been found to decrease the risk of myopia in non-myopic children and also slow down the progression of refractive errors and axial length in myopic children (13). Additionally, living environment (14), screen time (15) and physical characteristics (16) were associated with the prevalence of myopia. Furthermore, the link between education and myopia has become a key topic. The hypothesis that reducing the burden of education may help control the high prevalence of myopia has been supported by previous studies, although there was insufficient evidence (3, 8, 17).

Previous studies have reported that education achievement and education level were associated with myopia (8, 18). Apart from these two indicators, other educational factors were rarely studied. The purpose of this study was to investigate the relationship between the prevalence of myopia and various indicators associated with the educational environment, and identify effective school-based strategies for preventing myopia.

## 2. Methods

### 2.1. Ethics statement

This study design followed the tenets of the Declaration of Helsinki for biomedical research in human subjects. The study was approved by Hefei Institutes of Physical Science, Chinese Academy of Sciences Ethics Committee. Informed consent forms were issued to parents by the school and confirmed before the participation in the study.

### 2.2. Study population

**Figure 1:**
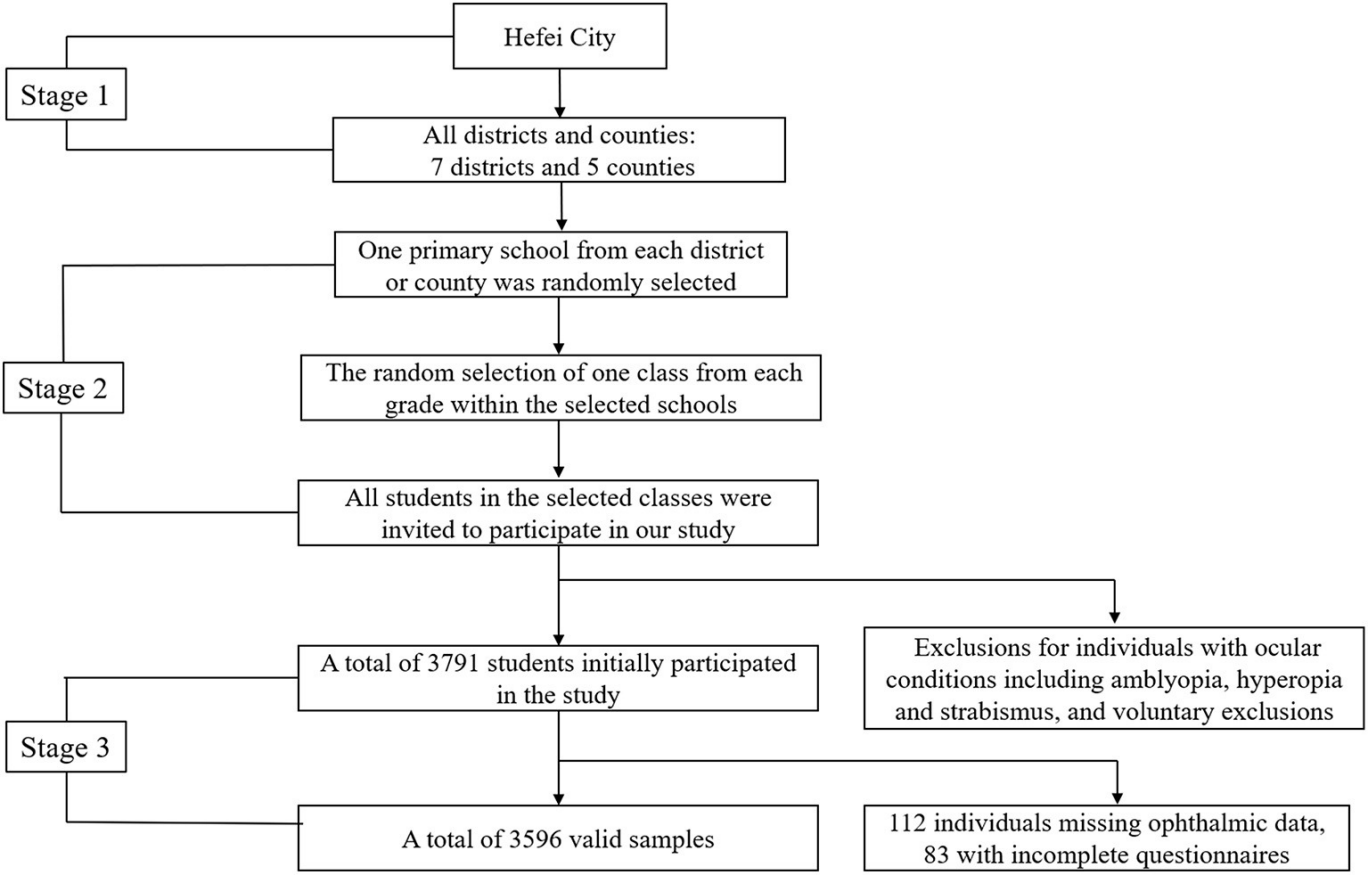
Flow diagram of multi-stage stratified cluster sampling.

This cross-sectional study was performed from July 2022 to October 2022 in Hefei, China. Hefei, the capital of Anhui Province, is located in the central part of China. A multi-stage stratified cluster sampling was used to obtain a representation of children (see Figure 1). There are a total of 7 districts and 5 counties in Hefei. One primary school was randomly selected from each district or county, followed by the random selection of one class from each grade within the selected schools. All students in the selected classes were invited to participate in our study. Individuals with other ocular conditions, including amblyopia, hyperopia and strabismus, were excluded, as well as those who decline to participate. Thus, a total of 3,791 students aged 6–14 initially participated in this study.

### 2.3. Assessment and definition of myopia

Similar to our previous study (19), a two-step ophthalmic examination was underwent. Firstly, children's visual acuity (VA) was examined by 5.0 logarithmic visual acuity chart light box according to the national standard rules (20). Then, individuals with VA worse than 5.0 (6/6 Snellen) in either eye without glasses underwent cycloplegic refraction, which was performed by ophthalmologists using 1% cyclopentolate eye drop. Those who had previously been diagnosed as myopia by cycloplegic refraction did not need to be tested again. All children with visual acuity < 6/6 Snellen in either eye without glasses and cycloplegic spherical equivalent refraction < -0.5 D were classified as myopic. Children with visual acuity ≥6/6 Snellen, or those with visual acuity < 6/6 Snellen, but cycloplegic spherical equivalent refraction ≥-0.5D, were classified as non-myopic.

### 2.4. Questionnaire

After the eye examination, the class teacher distributed a questionnaire to the parents through the smartphone. All children completed this questionnaire under the guidance of their parents. Demographic information was collected, including age, gender, grade and region. The response of region was divided into two options: city or county. The term “city” in this response specifically refers to the 7 districts situated in the central area of Hefei city. With regard to education environment of children, details were as follows (3, 21–23): parents' education level (the response categories: doctor or master, bachelor, below bachelor), children's academic level [excellent (grade A), good (grade B), qualified (grade C), unqualified (grade D)], parents' requirements for children's academic level (very high, high, general, low), the amount of children's homework per day (< 1 h, 1–2 h, 2–3 h, more than 3 h), after-school tutoring (none, 1–2 times per week, 3–4 times per week, more than 4 times per week), extracurricular reading (never, sometimes, often, always), the most frequent place to go on weekends (sports venues, leisure or entertainment places, learning places, staying at home) and taking programming classes (yes, no). Among of them, the amount of homework was assessed separately according to levels of school days and weekends. Children's academic levels were determined based on their performance in the most recent exam. Academic grades were adopted as a replacement for exam scores. Specifically, grade A refers to 90–100, grade B refers to 80–89, grade C refers to 60–79, and grade D is below 60 (the full score of 100).

### 2.5. Statistical analysis

The data was presented as number (%) for categorical variables, or mean (±standard deviation) for continuous variables. Differences in the distribution of variables between the non-myopia group and myopia group were assessed using the univariate analysis. All statistically significant factors (*p*-value < 0.05) in the univariate analysis were further analyzed by using a binary logistic regression model to determine the risk factors associated with myopia. These statistical analyses used the Statistical Package for Social Sciences (SPSS v22.0). Additionally, a random forest algorithm was applied to evaluate the importance of features. The random forest is a widely used classification modeling algorithm of machine learning, which uses decision trees as the base weak learner and adopt probabilistic decision rules. The five cross-validation and grid search were used to train the model and find optimal hyperparameters. All of the data was divided into five subsets. In each iteration, four subsets were used for training, and the remaining one was used for validation. The importance of features was based on a mean decrease impurity method (Gini importance) of tree model. These modeling analyses utilized the scikit-learn library, a Python-based machine learning toolkit.

## 3. Results

Of the initial 3,791 students, 112 individuals missed ophthalmic examination data, and 83 student questionnaires were incomplete. Finally, a total of 3,596 participants were involved in this analysis. Table 1 provides the distribution differences of the demographic information between myopia and no myopia group. Of the total group 27.1% (*n* = 974) children were myopic. The prevalence of myopia in boys was 24.9% and in girls was 29.5%, with a significant difference (*p* = 0.002). Myopia prevalence in students increased significantly with the grade level (*p* < 0.001). Specifically, the prevalence of myopia in grades 1–6 was 5.0, 10.2, 25.8, 34.2, 40.9, and 52.6, respectively. The myopia rate in the city and county was found to be nearly identical, and the difference between them was not considered statistically significant (*p* = 0.83).

**Table 1:** Subject demographic information.

**Variables**	**Total, *n* (%)**	**Myopia, *n* (%)**	**No myopia, *n* (%)**	***P***
**Gender**				0.002
Boy	1,871 (52.0)	465 (24.9)	1,402 (75.1)	
Girl	1,724 (48.0)	508 (29.5)	1,216 (70.5)	
**Grade**				< 0.001
1	437 (12.2)	22 (5.0)	415 (95.0)	
2	758 (21.1)	77 (10.2)	681 (89.8)	
3	779 (21.7)	201 (25.8)	568 (74.2)	
4	609 (16.9)	208 (34.2)	401 (65.8)	
5	572 (15.9)	234 (40.9)	338 (59.1)	
6	441 (12.3)	232 (52.6)	219 (47.4)	
**Region**				0.83
City	1,830 (50.9)	495 (27.0)	1,335 (73.0)	
County	1,766 (49.1)	479 (27.1)	1,287 (72.9)	
**Total**	3,596 (100%)	974 (27.1)	2,622 (72.9)	

Comparison of the educational environment information difference between myopia group vs. non-myopia group was shown in Table 2. Education level of the father, education level of the mother, academic level of children, hours of homework per day on school days, hours of homework per day on weekends, number of after-school tutoring per week, frequency of extracurricular reading, the most frequent place to go on weekends and taking programming classes distributions between myopia and non-myopia group were statistically significant different (*p* < 0.001). Additionally, although the univariate analysis showed a correlation between the parents' requirements for children's academic level and myopia, this was not statistically significant (p = 0.14).

**Table 2:** Univariate factor analysis of the risk of myopia.

**Variables**	**Total, *n* (%)**	**Myopia, *n* (%)**	**No myopia, *n* (%)**	***P***
**Education level of the father**				< 0.001
Doctor or master	68 (1.9)	32 (47.1)	36 (52.9)	
Bachelor	763 (21.2)	289 (37.9)	474 (62.1)	
Below bachelor	2,765 (76.9)	653 (23.6)	2,112 (76.4)	
**Education level of the mother**				< 0.001
Doctor or master	48 (1.3)	32 (66.7)	16 (33.3)	
Bachelor	575 (16.0)	210 (36.5)	365 (63.5)	
Below bachelor	2,973 (82.7)	732 (24.6)	2,241 (75.4)	
**Academic level**				< 0.001
Excellent (grade A)	479 (13.3)	207 (43.2)	272 (56.8)	
Good (grade B)	1,359 (37.8)	411 (30.2)	948 (69.8)	
Qualified (grade C)	1,260 (35.0)	267 (21.2)	993 (78.8)	
Unqualified (grade D)	498 (13.9)	89 (17.9)	1,016 (82.1)	
**Parents' requirements for children's academic level**				0.14
Very high	277 (7.7)	83 (30.0)	194 (70.0)	
High	1,547 (43.0)	433 (28.0)	1,114 (72.0)	
General	1,526 (42.4)	404 (26.5)	1,122 (73.5)	
Low	246 (6.8)	54 (22.0)	192 (78.0)	
**Hours of homework per day on school days**				< 0.001
More than 3 h	549 (15.3)	187 (34.1)	362 (65.9)	
2–3 h	1,072 (29.8)	310 (28.9)	762 (71.1)	
1–2 h	1,581 (44.0)	410 (25.9)	1,171 (74.1)	
< 1 h	394 (11.0)	67 (17.0)	327 (83.0)	
**Hours of homework per day on weekends**				< 0.001
More than 3 h	947 (26.3)	337 (35.6)	610 (64.4)	
2–3 h	1,205 (33.5)	355 (29.5)	850 (70.5)	
1–2 h	1,126 (31.3)	235 (20.9)	891 (79.1)	
< 1 h	318 (8.8)	47 (14.8)	271 (85.2)	
**Number of after-school tutoring per week**				< 0.001
>4 times	128 (3.6)	52 (40.6)	76 (59.4)	
3–4 times	459 (12.8)	169 (36.8)	290 (63.2)	
1–2 times	1,652 (45.9)	480 (29.1)	1,172 (70.9)	
0	1,357 (37.7)	273 (20.1)	1,084 (79.9)	
**Frequency of extracurricular reading**				< 0.001
Always	451 (12.5)	162 (35.9)	289 (64.1)	
Often	1,517 (42.2)	474 (31.2)	1,043 (68.8)	
Sometimes	1,565 (43.5)	332 (21.2)	1,233 (78.8)	
Never	63 (1.8)	6 (9.5)	57 (90.5)	
**The most frequent place to go on weekends**				< 0.001
Sports venues	650 (18.1)	152 (23.4)	498 (76.6)	
Leisure or entertainment places	529 (14.7)	114 (21.6)	415 (78.4)	
Learning places	1,026 (28.5)	332 (32.4)	694 (67.6)	
Staying at home	1,391 (38.7)	376 (27.0)	1,015 (73.0)	
**Taking programming classes**				0.003
Yes	546 (15.2)	176 (32.2)	370 (67.8)	
No	3,050 (84.8)	798 (26.2)	2,252 (73.8)	
Total	3,596 (100%)	974 (27.1)	2,622 (72.9)	

All statistically significant factors in the univariate analysis were incorporated into the multivariate logistic regression model and mutually adjusted. Results of adjusted logistic regression analysis were shown in Table 3. Boys were at a lower risk of myopia than girls (OR = 0.83, 95% CI = 0.70–0.98). The grade was the significant influencing factor, and ORs of grade 1–5 were 0.05 (95% CI = 0.03–0.08), 0.10 (95% CI = 0.07–0.13), 0.33 (95% CI = 0.26–0.43), 0.51 (95% CI = 0.39–0.66) and 0.63 (95% CI = 0.48–0.82), respectively. Parents with high education background tend to have children with myopia. For instance, students whose mothers are masters or doctors (OR = 4.89, 95% CI = 2.22–10.81) were more likely to have myopia than those whose mothers are below bachelor. Furthermore, the results demonstrated that academic level was significantly associated with the odds of myopia. Children with Grade A for academic achievement (OR = 2.95, 95% CI = 2.11–4.12), and with Grade B (OR = 1.98, 95% CI = 1.49–2.64), had the higher prevalence of myopia than children only achieved grade D in academic performance. With regard to homework load, spending more than 3 h (OR = 1.52, 95% CI = 1.01–2.29), or 2–3 h (OR = 1.39, 95% CI = 0.94–2.04) doing homework per day on weekends could increased the odds of myopia compared with < 1 h. The amount of homework per day on school days lost the statistical significance in the adjusted logistic regression model. The results also demonstrated that the association between after-school tutoring and the prevalence of myopia was significant. The myopia odds were respectively 1.86 times, 1.46 times higher among students taking more than 4 after-school tutoring per week (OR = 1.86, 95% CI = 1.22–2.85), 3–4 after-school tutoring per week (OR = 1.86, 95% CI = 1.11–1.92) compared to those having no after-school tutoring. Moreover, children who frequently extracurricular reading were more likely to have myopia than those who never read extracurricular books (always: OR = 2.92, 95% CI = 1.18–7.30; often: OR = 2.89, 95% CI = 1.19–7.01; sometimes: OR = 2.19, 95% CI = 0.91–5.33). In addition, there was no collinearity among the final statistically significant variables (VIFs: 1.00–1.52).

**Table 3:** Analysis of influencing factors of myopia based on logistic regression.

**Variables**	**AOR**	**95% CI**	***P***
**Gender**			
Boy	0.83	0.70–0.98	0.02
Girl			
**Grade**			
1	0.05	0.03–0.08	< 0.001
2	0.10	0.07–0.13	< 0.001
3	0.33	0.26–0.43	< 0.001
4	0.51	0.39–0.66	< 0.001
5	0.63	0.48–0.82	0.001
6			
**Education level of the father**			
Doctor or master	1.05	0.53–2.05	0.89
Bachelor	1.95	1.55–2.45	< 0.001
Below bachelor			
**Education level of the mother**			
Doctor or master	4.89	2.22–10.81	< 0.001
Bachelor	1.27	0.98–1.64	0.04
Below bachelor			
**Academic level**			
Excellent (grade A)	2.95	2.11–4.12	< 0.001
Good (grade B)	1.98	1.49–2.64	< 0.001
Qualified (grade C)	1.32	0.99–1.76	0.05
Unqualified (grade D)			
**Hours of homework per day on school days**			
More than 3 h	1.21	0.82–1.78	0.25
2–3 h	1.02	0.71–1.45	0.42
1–2 h	1.08	0.78–1.50	0.15
< 1 h			
**Hours of homework per day on weekends**			
More than 3 h	1.52	1.01–2.29	0.02
2–3 h	1.39	0.94–2.04	0.03
1–2 h	1.18	0.81–1.172	0.44
< 1 h			
**Number of after-school tutoring per week**			
> 4 times	1.86	1.22–2.85	0.004
3–4 times	1.46	1.11–1.92	0.007
1–2 times	1.15	0.95–1.39	0.16
0			
**Frequency of extracurricular reading**			
Always	2.92	1.18–7.30	0.02
Often	2.89	1.19–7.01	0.04
Sometimes	2.19	0.91–5.33	0.08
Never			
**The most frequent place to go on weekends**			
Sports venues	0.825	0.645–1.055	0.13
Leisure or entertainment places	0.918	0.700–1.204	0.54
Learning places	0.883	0.716–1.088	0.24
Staying at home			
**Taking programming classes**			
Yes	1.000	0.797–1.254	0.66
No			

**Figure 2:**
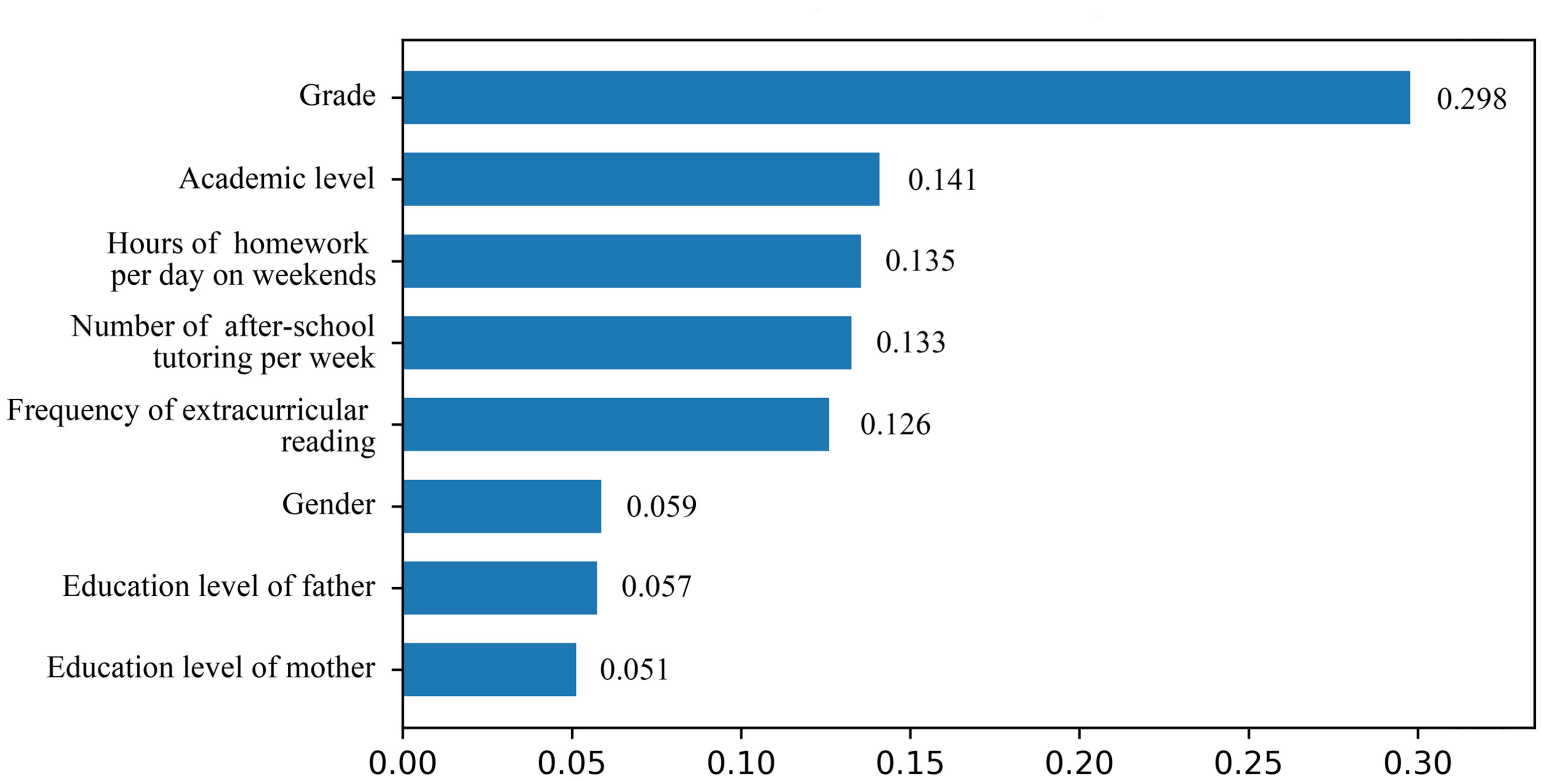
Feature importance based on the random forest model.

Furthermore, a random forest algorithm was used to classify samples based on these 8 significant variables associated with myopia, including gender, grade, education level of the father, education level of the mother, academic level of children, hours of homework per day on weekends, number of after-school tutoring per week and frequency of extracurricular reading. The result showed that these features can effectively identify children with myopia (Accuracy = 0.893). Figure 2 showed feature ranking in descending order of their importances calculated by the random forest model. Of the total feature set, the top 3 features were grade, academic level of children and hours of homework per day on weekends.

## 4. Discussion

This present study investigated the prevalence of myopia and evaluated the association of the educational environment with the myopia prevalence. The logistic regression model and the random forest model showed that educational load significantly increased the odds of myopia, especially heavy homework and extracurricular tutoring.

This study based on the stepwise ophthalmic examination indicated that the overall prevalence of myopia was 27.1% among primary school-aged students in Hefei, China. Although the prevalence of myopia in students in grade 1 was only 5.0%, it increased to 25.8% in students in grade 3. More than half of the students in grade 6 were myopia. This prevalence rate was lower than that reported by the National Health Commission (2). The main reason may be that the goal of the National Health Commission was to screening myopia, which only used the visual acuity chart in many cases (24). Results also revealed that girls were more likely to be myopic than boys. According to previous studies, this phenomenon was common in children of different regions or ages (3, 4, 25).

Our study has demonstrated that educational environment was significantly associated with the prevalence of myopia. As argued by Morgan et al., the tendency for schooling to lead to increased myopia have been documented in almost all major population groups (8, 23, 26). In terms of specific factors, better academic performance seemed to be accompanied by higher prevalence of myopia. Similarly, the Organization of Economic Cooperation and Development (OECD) Program in Secondary Assessment (PISA) surveys showed that students from countries with high myopia rates such as China, Japan, and Singapore, have achieved leading positions in various educational outcomes such as science and reading, and the high educational achievement and high myopia prevalence in developed countries in East and Southeast Asia were interrelated (18). This result was also similar with Wang's study based on the visual acuity showing that students with poor vision were more likely to have better mathematics achievement than those with normal vision (27). However, the previous study in Guangzhou, China, did not demonstrate any significant association between school academic achievement and myopia (28).

Compared with other educational factors, the effect of the homework load on myopia has been widely concerned. The association between average time spent on homework per day and myopia has been consistently observed in recent years, especially in China (27, 29, 30). Our study first investigated the homework loads on weekends and school days respectively, and evaluated their impacts on the prevalence of myopia. After incorporating all the significant variables and mutually adjusting for covariates, the final model showed that only the amount of homework per day on weekends was associated with the odds of myopia, whereas that on school days was not. Furthermore, the amount of homework per day on school days lost statistical significance after only adjusting for grade. This finding was important, showing that reducing weekend homework amount may be the most immediate path to preventing myopia.

Moreover, this study indicated that more after-school tutoring was significantly associated with the higher odds of myopia. The result was consistent with Tsai's study in Taipei showing that participants who spent ≥5 h every week on afterschool tutoring programs had greater risk for incident myopia (31). The Zhao's study also reported that often taking extracurricular tuition was more likely to be associated with myopia (32). Extensive use of afterschool tutorials has been suggested as a marker of educational environments which impose high educational loads (18). Compared with locations with high educational performance and low myopia rate, locations with high educational performance and high myopia rate have a high engagement in after-school tutorials (18).

The results showed that frequently extracurricular reading led to a higher risk of myopia. Similarly, two prior studies have also demonstrated that reading for pleasure was positively associated with myopia (10, 28). In addition, our study found that there was a trend for higher myopia prevalence among children with parents of higher level of education, which was consistent with the previous study (33). It is postulated that the association between parental education and myopia in the child may be due to these parents having myopia (as a result of their time studying/education) and the child having a genetic predisposition. Also, the level of parents' education may effect parents' awareness for myopia and family health education.

Furthermore, the feature importance analysis based on a random forest revealed that, in addition to grade, academic level of children and hours of homework per day on weekends were the most crucial factors, followed by number of after-school tutoring per week. This feature importance was found to be correlated with statistical significance in both univariate analysis and multivariate analysis. The ranking of importance appeared to follow the trend of p-values. For instance, the variable ‘grade' and ‘academic level' held very low p-values (< 0.001), and were ranked as highly important in this random forest model.

## 5. Limitations

Some methodological limitations need be considered. First, this study only examined factors associated with the educational environment and did not account for other prevalent risk factors of myopia, such as parental myopia and time outdoors. Second, this study used a stepwise ophthalmic examination. Cycloplegic autorefraction was obtained only in those children with visual acuity of < 5.0 in either eye. Those children with normal visual acuity could still be myopic. Additionally, responses to the questionnaire were self-reported, which may be a source of recall bias.

## 6. Conclusions

The current study showed that educational environment was strongly associated with the prevalence of myopia, including education level of the father, education level of the mother, academic level of children, hours of homework per day on weekends, number of after-school tutoring per week and frequency of extracurricular reading. Among them, homework on weekends and after-school tutoring had the strongest association with myopia, and were also the easiest to modify. Lightening the burden of studying may be the most immediate path for myopia prevention.

## Data availability statement

The raw data supporting the conclusions of this article will be made available by the authors, without undue reservation.

## Ethics statement

The studies involving human participants were reviewed and approved by Hefei Institutes of Physical Science, Chinese Academy of Sciences Ethics Committee. Written informed consent to participate in this study was provided by the participants' legal guardian/next of kin.

## Author contributions

Conceptualization: WP and SS. Data curation: WP and ZZ. Investigation: WP and FW. Formal analysis and writing—original draft: WP. Project administration and writing—review and editing: SS and YS. Resources: FW and SS. All authors contributed to the article and approved the submitted version.
